# The history of giardiasis

**DOI:** 10.1007/s00436-025-08607-9

**Published:** 2025-12-10

**Authors:** Dietmar Steverding

**Affiliations:** Bob Champion Research and Education Building, Norwich Medical School, University of East Anglia, Norwich Research Park, Rosalind Franklin Road, Norwich, NR4 7UQ UK

**Keywords:** Giardia duodenalis, G. intestinalis, G. lamblia, Giardiasis, History

## Abstract

This review paper outlines the history of giardiasis caused by *Giardia duodenalis*. Discovered in 1681, *G. duodenalis* is probably the first protozoan parasite ever observed by a human. Despite the early discovery, the taxonomic status of the protozoan remained uncertain for a long time. The reason for this is that *G. duodenalis* is a species complex comprising several phylogenetically distinct genotypes termed assemblages. Initially, it was thought that *G. duodenalis* is a primitive eukaryote because it lacks many subcellular organelles. However, recent research has shown that the protozoan has diverged from higher eukaryotes and that the lack of subcellular organelles is due to secondary loss and reduction. Based on paleoparasitological findings, *G. duodenalis* has parasitised humans since antiquity and has probably been spread globally by human migratory activity. Interestingly, it was not until 1987 that the pathogenicity of *G. duodenalis* was demonstrated for humans. Giardiasis is still a public health problem in the 21st century, particularly in young children living in areas with low hygiene standards.

## Introduction

*Giardia duodenalis* (syn. *G. intestinalis* and *G. lamblia*) is a protozoan parasite causing enteric infections and diarrhoea in humans and animals. The diarrhoeal illness, known as giardiasis, occurs globally, and in 2010, the World Health Organization (WHO) estimated 183,842,615 (95% Uncertainty Interval (UI): 130,018,020 to 262,838,002) cases of the disease (Torgerson, et al., 2015). Giardiasis is not a fatal infection but in malnourished and dehydrated infants, it can contribute to death (Anetor and Ogungbemi 2009). The disease is a very common intestinal protozoal infection worldwide, and children under 5 years are the most affected age group (WHO 2025). Usual symptoms of clinical manifested giardiasis are watery diarrhoea and flatulence, but asymptomatic courses of the infection also occur. The infection typically last for several weeks (2–6 weeks) and can be chronic or recurring (WHO 2025).

Humans and animals get infected with *G. duodenalis* by ingesting cysts in contaminated water or food, or by direct person-to-person contact via the faecal-oral route (Dunn and Juergens 2024). The cysts of *G. duodenalis* are robust and can survive several weeks to months in the environment, especially in cold and damp conditions (Huang and White 2006). When reaching the small intestine, each cyst releases an excyzoite containing four nuclei which subsequently divides twice into four trophozoites each containing two nuclei (Bernander et al. 2001). The trophozoites replicate rapidly by binary fission along the longitudinal axis. They can attach to the intestinal wall via a ventral sucking disk or swim freely in the intestinal lumen. After transit towards the colon, the trophozoites are converted back into cysts, which are excreted in diarrhoeal and non-diarrhoeal faeces. Trophozoites can also be found in loose stools, but this is not common. The trophozoites are diagnostically identifiable as pear-shaped flagellates with two nuclei.

The exact pathophysiological mechanism of giardiasis is still not fully understood. It has been found that the parasite induces morphological changes to the microvilli and disrupts epithelial cell junctions, resulting in increased intestinal permeability (Buret 2008; Koh et al. 2013). The colonisation of the small intestine by *G. duodenalis* has also been shown to induce apoptosis in the intestinal epithelial cells (Koh et al. 2013). These pathologies may result in an altered gastrointestinal motility (Dunn and Juergens 2024). The infection with *G. duodenalis* also results in a decreased expression of brush border enzymes, causing malabsorption of nutrients and electrolytes (Buret 2008; Cotton et al. 2011). As a consequence, an osmotic gradient is created inducing water to be drawn into the lumen of the small intestine. Due to the influx of water, the small intestine distends and rapidly contracts. This can increase the intestinal transit rates intensifying diarrhoeal symptoms in patients infected with *G. duodenalis*. In addition, giardiasis can also cause hypersecretion of chloride that further contributes to watery diarrhoea. Moreover, giardiasis patients commonly have elevated levels of fatty acids in their stool (Santos-Martins et al. 2024). This steatorrhoea is probably cause by the impaired breakdown of carbohydrates that are subsequently converted into short-chain fatty acids by colonic microbiota (Robayo-Torres et al. 2006). Together, these pathologies lead to malnutrition and weight loss, particularly in children in low- and middle-income countries, with the long-term consequences of growth and cognitive development impairment (Gutiérrez and Bartelt 2024). In addition, there is evidence that *G. duodenalis* alters the gut microbiome, which could change the intestinal homeostasis, reduce the intestinal microbial diversity, and lead to the overexpression of pathogenic and proinflammatory bacteria (Fekete et al. 2021). In combination with polymicrobial infections, *G. duodenalis*-infection may exacerbate stunted growth in malnourished children. Additionally, giardiasis can also cause immune system disorders that may vary depending on the patient’s age and comorbidities (Klimczak et al. 2024). For instance, the attachment of the parasite to the intestinal wall leads initially to an inflammatory response by the host to control the infection. However, in chronic infection, the inflammatory response can be weakened to a state of immune tolerance helping the parasite to survive. Moreover, studies have shown that infections with *G. duodenalis* are associated with an increased risk of developing irritable bowel syndrome (Nakao et al. 2017; Abedi et al. 2022).

This review article is focussed on historical aspects of giardiasis discussing the evolution of *G. duodenalis*, the palaeoparasitological evidence of giardiasis, the discovery of *Giardia* sp., outbreaks of the recent past, the establishment of *G. duodenalis* genetic assemblages, the development of currently approved giardiasis treatments, and the current epidemiological situation of the diseases. New developments like the use of organoids to investigate the pathogenesis of giardiasis, current assemblage typing procedures, or compound screening to identify new drugs against *G. duodenalis* are not reviewed in this article.

## Evolution of *G. duodenalis*

As *Giardia* species lack mitochondria, peroxisomes/microbodies, normal endoplasmic reticulum (ER), and Golgi, they were previously thought to be primitive extant eukaryotes that branched off from other eukaryotes before the canonical eukaryotic organelles had evolved (Keeling 1998; Lloyd and Harris 2002). However, cytological studies and genome sequencing revealed that G. *duodenalis* has remnants and many genes for the “missing” organelles (Lloyd and Harris 2002; Morrison et al. 2007). Already in the 1960 s, filamentous structures and subpellicular bodies were observed in cysts and trophozoites of G. *duodenalis* by light and electron microscopy, respectively, which were thought to be mitochondria (Nath and Dutta 1962; Cheissin 1965). By using the membrane-potential-sensitive dye rhodamine 123 and fluorescence microscopy, it was later shown that the parasite has membranous structures with membrane-potential-generating and electron transport functions (Lloyd et al. 2002), features that are characteristic of mitochondria. Localisation studies of the mitochondrial marker proteins IscU and IscS revealed the existence of double-membraned mitochondrion-like organelles (mitosomes) in *G. duodenalis* that play an essential role in iron-sulphur protein maturation (Tovar et al. 2003). In addition, three typical mitochondrial genes encoding for the valyl-tRNA synthetase, the mitochondrial-like chaperonin 60 (Cpn60), and the mitochondrial-type heat shock protein 70 (HSP70) have been detected in the nuclear genome of *G. duodenalis* (Hashimoto et al. 1998; Roger et al. 1998; Morrison et al. 2001). Similarly, the two peroxisomal proteins acyl-coenzyme A synthetase long-chain family member 4 (ACSL-4) and peroxin-4 (PEX-4) have been localised in cytoplasmic vesicles of trophozoites (Acosta-Virgen et al. 2018), suggesting the presence of peroxisome-like organelles in *G. duodenalis*. Although a recognisable Golgi complex has not yet been identified in *G. duodenalis*, using the Golgi apparatus fluorescent probe C6-NBD ceramide, Golgi membranes in the perinuclear regions have been detected in trophozoites (Lanfredi-Rangel, et al., 1999). In addition, the parasite has the protein complexes COPI and COPII (Marti et al. 2003) that usually facilitate retrograde and anterograde transport between the Golgi and ER in higher eukaryotes. Furthermore, the adaptor protein complex 1 was also discovered in *G. duodenalis*, which is involved in the anterograde protein trafficking to peripheral vacuoles in the parasite (Touz et al. 2004). The presence of ER in *G. duodenalis* was demonstrated with antibodies to the ER-protein BiP, which identified ER cisternae and tubules, and stacked perinuclear membranes (Soltys et al. 1996). More recent research found that the ER in *G. duodenalis* is mainly arranged as a tubulovesicular network (Abodeely et al. 2009). All of this suggests that *G. duodenalis* once possessed classical eukaryotic organelles that were lost through evolutionary reduction.

Like many obligate parasitic organisms, *G. duodenalis* has reduced biosynthetic and energy pathways (Adam 2001, 2021). As the parasite lacks cytochromes and thus oxidative phosphorylation, the energy production is entirely through fermentative metabolism. Glucose is the major energy source, which is metabolised into acetate, ethanol, alanine, and CO_2_, depending on the oxygen concentration (Paget et al. 1990). Moreover, two key enzymes of the glycolytic pathway of *G. duodenalis* are pyrophosphate-dependent, i.e., PPi-dependent phosphofructokinase (PPi-PFK) (Mertens 1990) and pyruvate phosphate dikinase (PPDK) (Hrdý, et al., 1993). The use of pyrophosphate-dependent glycolytic enzymes seems to improve the energy efficiency of glycolysis (Mertens 1993). In addition to generating energy by fermenting glucose, the metabolism of amino acids provides important energy production pathways in *G. duodenalis* (Adam 2001). In particular, the amino acid arginine can be converted via the arginine dihydrolase pathway into ornithine and one molecule of ATP (Schofield et al. 1992). Furthermore, *G. duodenalis* does not have the capacity of de novo synthesis of purine and pyrimidine nucleotides and therefore is dependent on salvage pathways to obtain these nucleoside phosphates (Adam 2001). Also, the parasite has a limited lipid synthesis ability (Das et al. 2002) and seems to satisfy its lipid requirement by taking up cholesterol and phosphatidylcholine from the external environment (Lujan et al. 1996). Notably, *G. duodenalis* cannot synthesise glycerophospholipids de novo but is able to remodel exogenous glycerophospholipids or to use intermediate dietary products of the host to produce some glycerophospholipids (Ye et al. 2017). The parasite can do this through simple and incomplete glycerophospholipid pathways, characterised by loss of genes and horizontal transfer of bacterial genes (Ye et al. 2017). The many reduced and missing biosynthesis pathways may indicate that *G. duodenalis* is an early and primitive eukaryote. On the other hand, it is also obvious that many of the biosynthesis pathways have undergone secondary adaptation to the parasitic lifestyle of the organism.

**Fig. 1:**
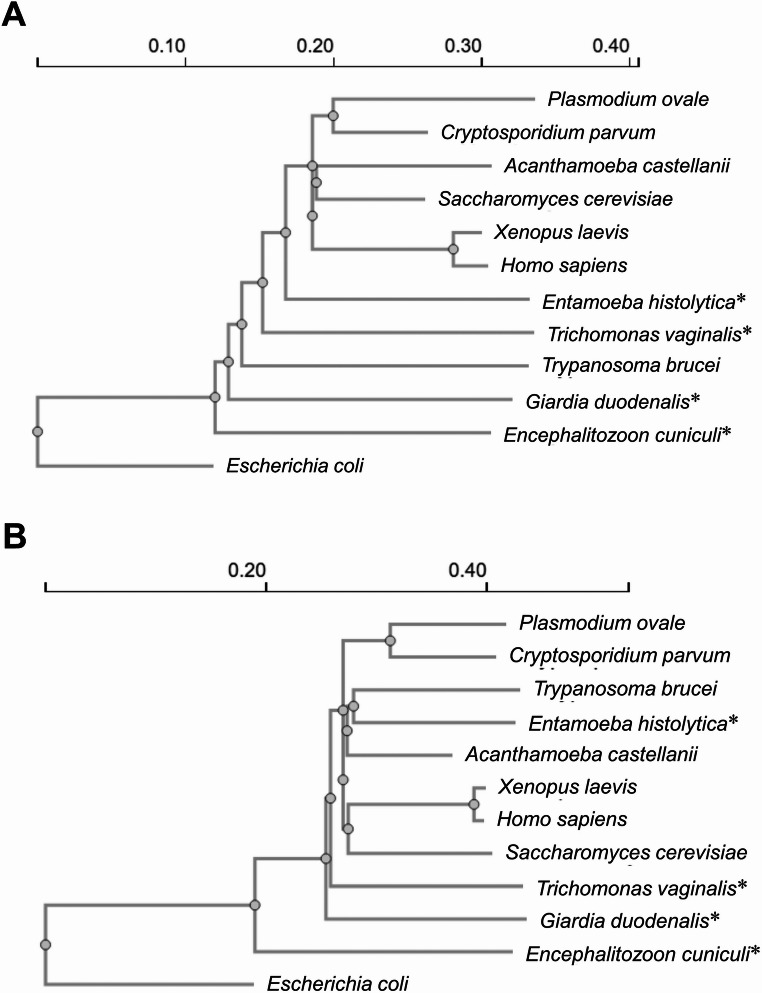
Small subunit ribosomal RNA (**A**) and elongation factor 2 (**B**) phylogenetic trees of eukaryotes with *Escherichia coli* as an outgroup species. The phylogenetic dendrograms were calculated using the neighbour-joining method with the programme Clustal Omega (Madeira et al. 2024). Asterisks (*) denote amitochondriate eukaryotes

Previous phylogenetic analyses of small subunit ribosomal RNAs and elongation factors 1 and 2 place *G. duodenalis* at the base of the eukaryotic tree (Sogin 1989; Sogin et al. 1989; Hashimoto et al. 1994, 1995). In contrast, phylogenetic analysis of β-tubulin sequences revealed that *Entamoeba histolytica* represents the earliest offshoot among eukaryotes, and *G. duodenalis* the fourth earliest offshoot (Edlind et al. 1996). However, reanalysis of small subunit ribosomal RNA and elongation factor 2 sequences using the Clustal Omega multiple sequence alignment programme showed that the microsporidian *Encephalitozoon cuniculi* represents the deepest branching lineage in the eukaryotic tree, with *G. duodenalis* being the second earliest offshoot (Fig. 1). To determine the exact position of *G. duodenalis* within the eukaryotic tree, further phylogenetic analysis of genes encoding conserved proteins are required.

## Historical evidence of *G. duodenalis*

### Prehistory (before 3200 BCE)

**Fig. 2:**
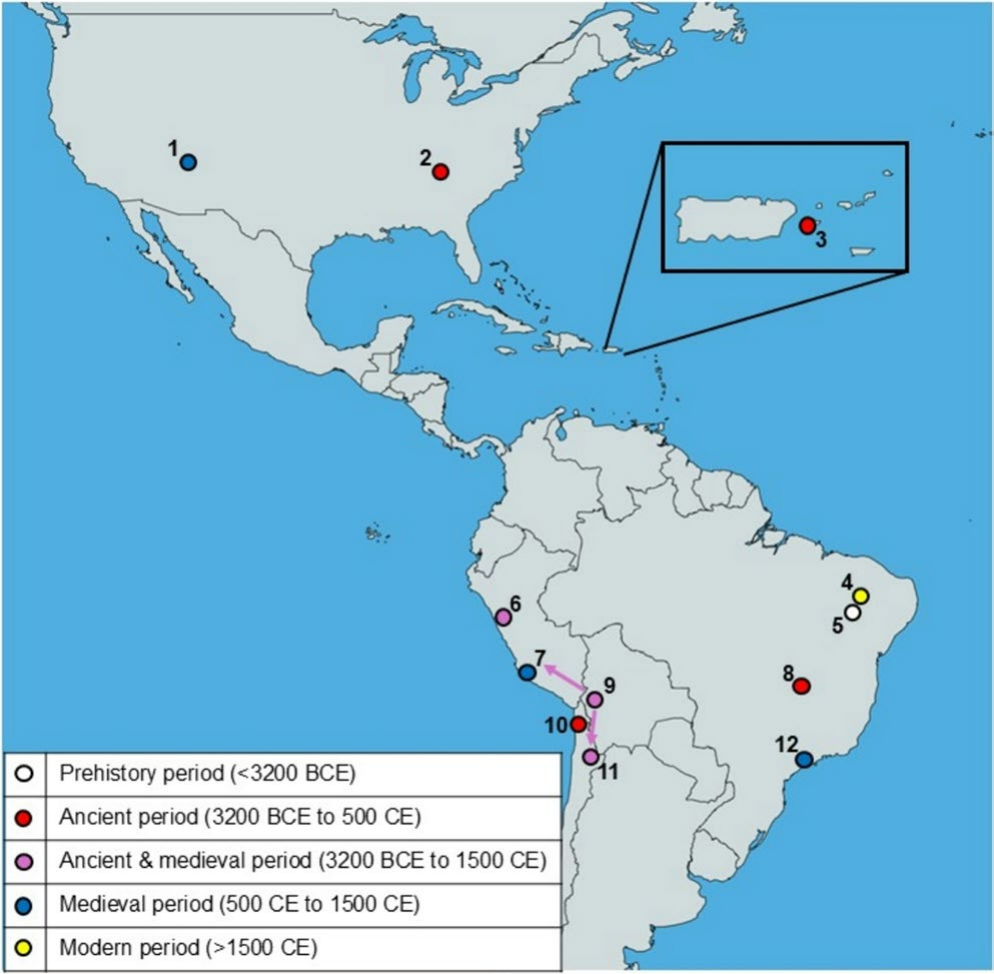
Locations of historical evidence of *G. duodenalis* in the New World. 1, Canyon De Chelly; 2, Big Bone Cave; 3, Island of Vieques; 4, Toca da Baixa dos Caboclos; 5, Boqueirão da Pedra Furada; 6, Los Gavilanes; 7, Huayuri; 8, Gruta do Gentio; 9, South American Andes; 10, Tarapacá 40 cemetery; 11, San Pedro de Atacama, 12, Fonseca. The map template is from MapCharts: https://www.mapchart.net

The earliest evidence of *G. duodenalis* infections in humans comes from the New World from the archaeological site of Boqueirão da Pedra Furada located in the state of Piauí, Brazil (Fig. 2). Four human coprolites, 9800 to 7230 years old (7850 to 5280 BCE), were found positive for *Giardia* sp. surface antigen by enzyme-linked immunosorbent assay (ELISA), and one of the four specimens was also positive by immunofluorescence assay (IFA) (Leles et al. 2019).

### Ancient history (3200 BCE to 500 CE)

The first documentation of *Giardia* sp. detected in an ancient specimen was published by Faulkner et al. (1989) (Fig. 2). Protozoan-like cysts were discovered in human desiccated faecal material obtained at the Big Bone Cave excavation site in Tennessee, USA. The faecal specimen had a radiocarbon age of 2550 years (ca. 550 BCE) (Faulkner 1991). The observed cysts had ellipsoid shapes and were identified by IFA as *Giardia* sp. A second report describes the identification of *Giardia* sp. in faeces from the intestines of 500 to 3000-year-old Andean mummies (ca. 1000 BCE to 1500 CE) (Allison et al. 1999) (Fig. 2). Of the 39 specimens, 9 gave a positive result by IFA. However, since the specimens were not assigned to a specific age, it remains unclear which of the positive samples dates back to ancient times. A third description of the ancient presence of *Giardia* sp. comes from a coprolite collected from an archaeological site at the Los Gavilanes preceramic settlement in Peru (Ortega and Bonavia 2003) (Fig. 2). The coprolite was radiocarbon-dated to be 3525–4375 years old (ca. 2375 − 1525 BCE). The cysts enclosed in the coprolite were recognised as *Giardia* sp. by IFA. A fourth report describing the identification of *Giardia* sp. in human coprolites by ELISA was published 2019. The specimens were recovered at three different excavation sites: Gruta do Gentio, state of Minas Gerais, Brazil (three coprolites dating back to 1540 BCE); Tarapacá 40 cemetery, Caserones, Chile (one coprolite dating back to 900 BCE to 800 CE); and San Pedro de Atacama, Chile (one coprolite dating back to 100–500 CE) (Leles et al. 2019) (Fig. 2). A fifth account of *G. duodenalis* in an ancient specimen originates from an archaeological site at the Sorcé settlement in the Caribbean Island of Vieques, Puerto Rico (Wiscovitch-Russo et al. 2020) (Fig. 2). In this case, *G. duodenalis* was identified in a 1600 to 1800-year-old coprolite (ca. 200–400 CE) by metagenomic sequencing.

**Fig. 3:**
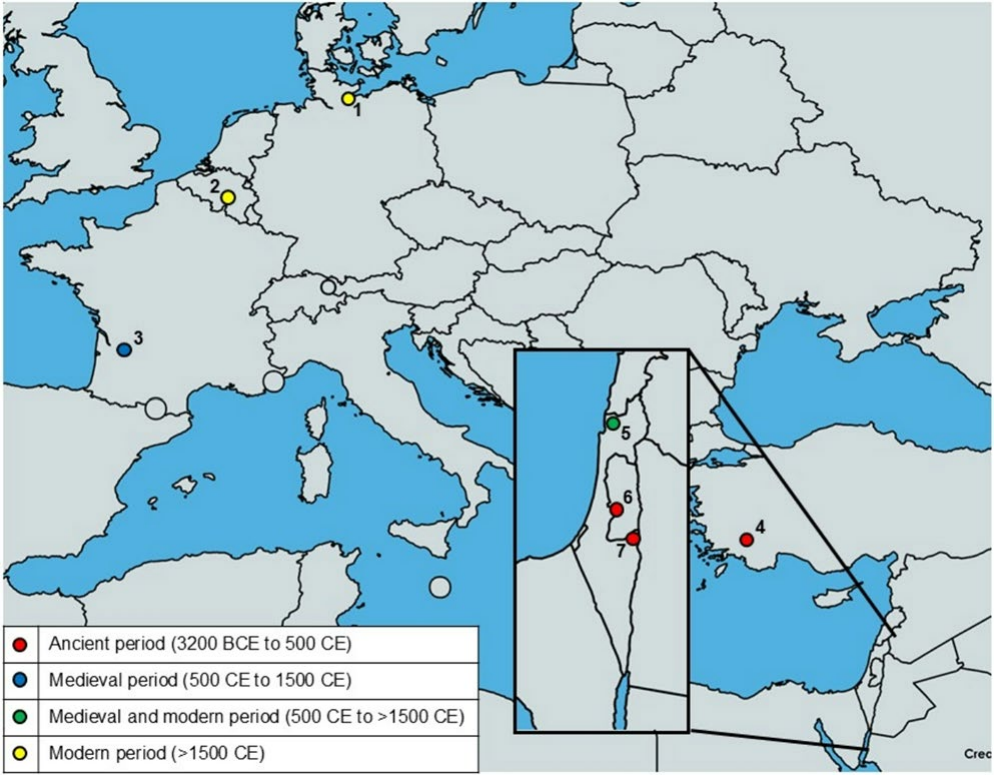
Locations of historical evidence of *G. duodenalis* in the Old World. 1, Lübeck; 2, Namur; 3, La Mothe de Pineuilh; 4, Sagalassos; 5, Acre; 6, Jerusalem; 7, Nahal Mishmar. The map template is from MapCharts: https://www.mapchart.net

There are three references to the ancient occurrence of *G. duodenalis* in the Old World (Fig. 3). The first report describes the microscopical identification of *G. duodenalis* cysts in two 1800-year-old human coprolites recovered from a cave near the Nahal Mishmar river in the Judean Desert in Israel (Witenberg 1961). However, since it was mentioned that the cysts in the specimens were not well preserved, it remains speculative whether the observed structures were indeed the dispersal stage of *G. duodenalis*. The second account comes from a study investigating the sediments of two latrines in Jerusalem dating from the 7th century BCE and 7th −6th century BCE, respectively (Mitchell et al. 2023). Rather than visualising cysts, an ELISA was used to detect *G. duodenalis* antigens in the sediments. Positive results were found for both latrines, indicating that *G. duodenalis* was present in the Near East before the Roman period. The third record is from the latrines of a Roman bath complex at Sagalassos, Türkiye, dating back to the 2nd to 5th century CE (Williams et al. 2017). In one out of five samples, *G. duodenalis* cysts were identified by ELISA.

### Middle ages (500 CE to 1500 CE)

There is some evidence for the occurrence of *G. duodenalis* in the Old World during medieval times (Fig. 3). In a first study, organic sediment layers from the archaeological site at La Mothe de Pineuilh in France, dating back to the 10th to 11th century CE, were investigated for the presence of *G. duodenalis* antigen (Le Bailly et al. 2008). Of nine samples, one was positive for the parasite antigen by IFA and ELISA. Two more records on the identification of *G. duodenalis* in sediments of medieval latrines come from two excavation sites in Israel: one in the City of Acre, radiocarbon dated to the 13th century CE (Mitchell et al. 2008), and the other one in Jerusalem assigned to the 15th century CE (Yeh et al. 2015). In the first case, one out of eight samples, and in the second case, one out of six samples, gave a positive result by ELISA.

There are a few published accounts of *G. duodenalis* recovery in medieval samples from the New World (Fig. 2). One of the reports comes from the excavation site of Los Gavilanes in Peru, where *G. duodenalis* had already been detected in ancient specimens (see chapter above) (Ortega and Bonavia 2003). In a human coprolite dating to the Peruvian Middle Horizon period (ca. 500–900 CE), cysts of *G. duodenalis* were found by IFM. This finding suggests that giardiasis was prevalent at the Los Gavilanes preceramic settlement for a period of several thousand years. The report on the Andean mummies also contains evidence of the medieval occurrence of *G. duodenalis* (see chapter above) (Allison et al. 1999). However, it is not clear how many of the positive specimens are from medieval times. Another account describes the detection of *G. duodenalis* in a human coprolite collected from a cesspit at the Canyon De Chelly archaeological site in Arizona, USA (Gonçalves et al. 2005). A parasite-specific antigen was detected in the specimen dating to about 1200 CE by ELISA. Leles and coworkers report the detection of *G. duodenalis* in specimens from sites in Brazil, Chile, and Peru (Leles at al. 2019). Four human sediment samples dating back to 950 CE from the Fonseca archaeological site, São Paulo, Brazil, were positive for *Giardia* sp. surface antigens. Two coprolite specimens dated to 900–1450 CE recovered at San Pedro de Atacama, Chile, gave also a positive result by ELISA. At the large pre-Columbian archaeological site of Huayuri, Peru, one human coprolite from 1200 to 1400 CE gave a positive ELISA result for *Giardia* sp. antigen.

### Modern times (1500 CE to present)

There is only one report on the discovery of *G. duodenalis* in a coprolite sample from the New World dating from modern times (Fig. 2). This specimen came from the archaeological site of Toca da Baixa dos Caboclos, state of Piauí, Brazil, and was determined to be 400 years old (1550 CE) (Leles et al. 2019).

Three accounts describe the identification of *G. duodenalis* in modern-time specimens from the Old World (Fig. 3). Soil samples from cesspits in Lübeck (Germany) and Namur (Belgium) dating back to the 17th and 18th century CE, respectively, tested positive for the presence of *G. duodenalis* antigen by ELISA (Gonçalves et al. 2005). Likewise, a sediment sample from a cesspit located in the old city of Acre, Israel, and dated to the early 1800 s (Ottoman Period), gave a positive ELISA result (Eskew et al. 2019).

**Fig. 4:**
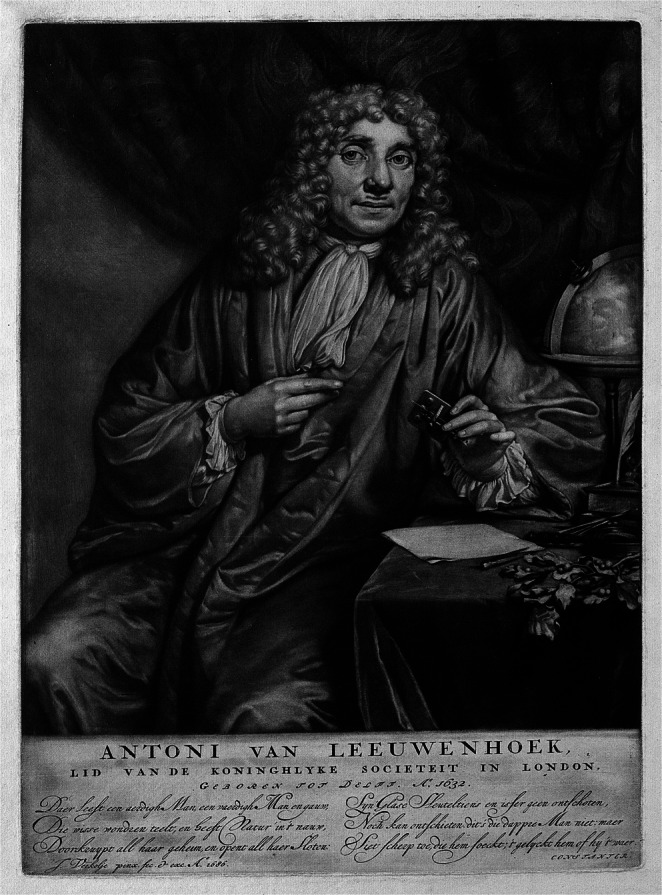
Antonie van Leeuwenhoek. Mezzotint by J. Verkolje, 1686. Wellcome Collection 5492i. Creative Commons CC-BY-4.0 Licence. Link: https://wellcomecollection.org/works/u4naz3q3

The first person to have seen trophozoites of *G. duodenalis* was most likely the Dutch microbiologist and microscopist Antonie Philips van Leeuwenhoek (1632–1723) (Fig. 4), when he examined his own diarrhoeal stool with a self-built, single-lens microscope device. Van Leeuwenhoek described his observation of the parasite in a letter dated November 4, 1681, to the English polymath Robert Hooke (1635–1703). Since the letter was written in early modern Dutch, it was translated into English and presented to the fellows of the Royal Society at a meeting held on November 9, 1681. The text passage concerning the description of *G. duodenalis* reads in the translation by the Committee of Dutch Scientists as follows (for the original Dutch text, see Box 1):

“*All the afore-said particles lay in a clear*,* transparent matter*,* in which clear matter I have sometimes seen animalcules moving very prettily*,* some of them a little bigger*,* others a little smaller than a globule of blood*,* but all of one and the same shape. Their bodies were a little longer than broad*,* and their belly was flattish and furnished with several legs*,* with which they moved through the clear matter and among the globules in such a manner that one might imagine seeing a wood-louse running up against a wall; and although they made a quick motion with their legs they for all that made but slow progress.*” (Commissie van Nederlandse 1948).

**Fig. 5:**
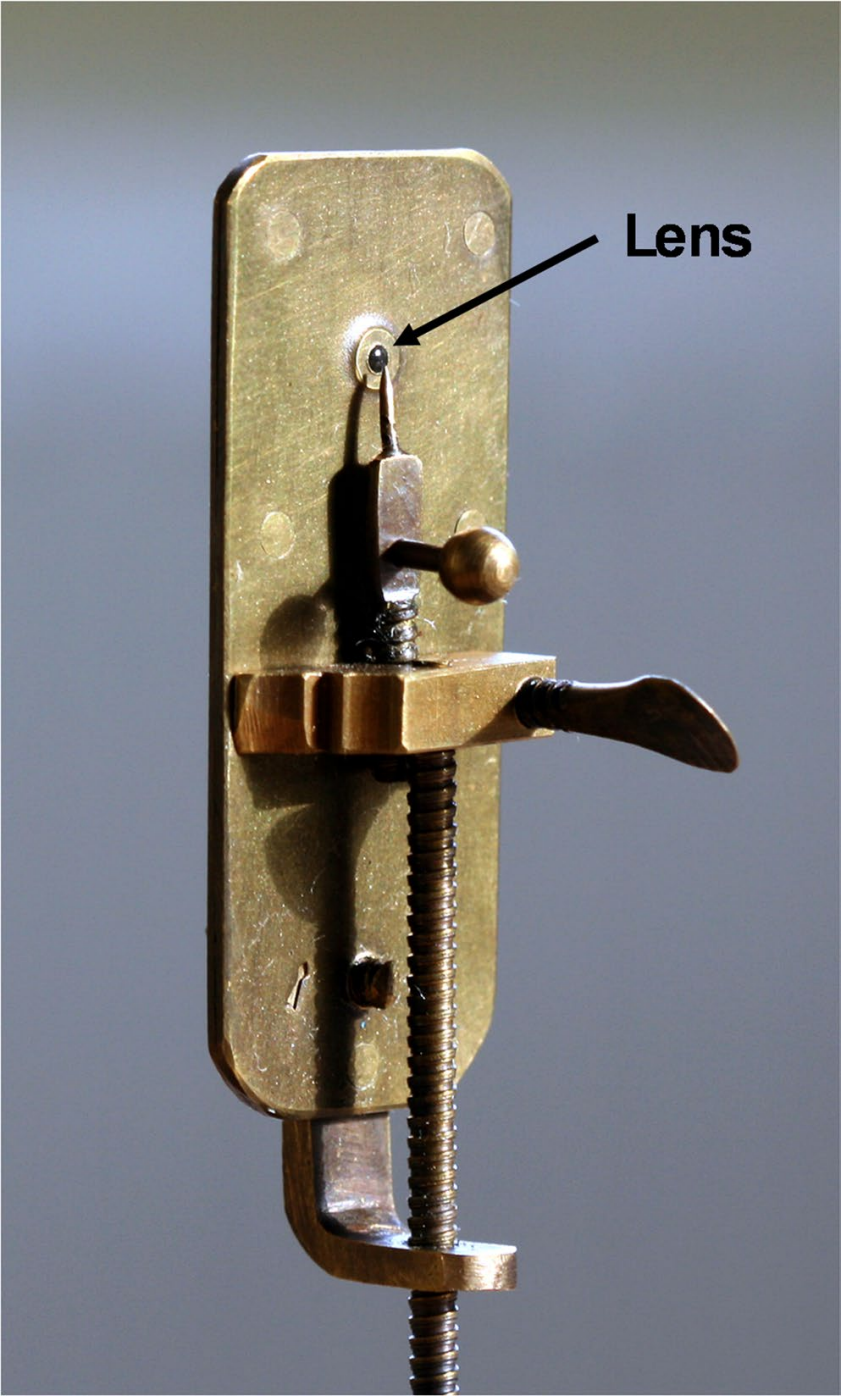
Replica of an Antonie van Leeuwenhoek’s single-lensed microscope. Photo by Jeroen Rouwkema, Creative Commons CC BY-SA 3.0 Licence. Link: https://creativecommons.org/licenses/by-sa/3.0 (via Wikimedia Commons)

Although van Leeuwenhoek’s description of the morphology and movement of the observed organism is recognisable as that of *G. duodenalis*, it was later often doubted whether the Dutchman had actually been able to see the protozoan with his primitive single-lensed microscope. In 1920, the British protozoologist Clifford Dobell (1886–1949) published a monograph in which he thoroughly analysed van Leeuwenhoek’s account and invalidated the counterarguments of the critics, concluding that van Leeuwenhoek’s description of the flagellate was unmistakable (Dobell 1920). Eventually, the British independent research biologist, author, and lecturer Brian Ford could demonstrate that it was possible to observe *G. duodenalis* with a replica of van Leeuwenhoek’s microscope (Fig. 5) (Ford 2005). Thus, it can be assumed with sufficient certainty that van Leeuwenhoek had indeed seen *G. duodenalis* almost 350 years ago.

**Fig. 6:**
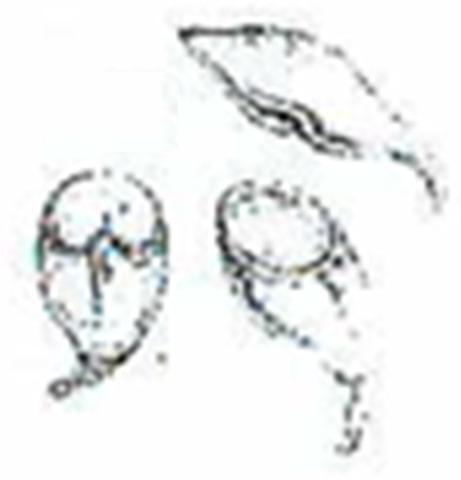
First microscopic drawing of *G. duodenalis* by the Bohemian physician Wilhelm Lambl. The characteristic appearance of the parasite is clearly recognisable. Reproduced from Lambl (1859). As the work is in the Public Domain (author’s life plus 70 years), no permission for reproduction is required

The next scientific reference to *G. duodenalis* was in 1859 when the Bohemian physician Vilém Dušan Lambl (1824–1895) (he authored his publications as Wilhelm Lambl) published an account on the presence of monads in mucous stools of children (Lambl 1859). The microscopic drawings (Fig. 6) disclosed with the study showed beyond doubt that the flagellates observed by Lambl were *G. duodenalis*. Lambl named the protozoan *Cercomonas intestinalis*, as it resembles other species of the genus *Cercomonas*. However, the species name *C. intestinalis* had already been in use when the kinetoplast *Bodo intestinalis* was transferred into the genus *Cercomonas* in 1850. In 1875, the French physician and microbiologist Casimir-Joseph Davaine (1812–1882) described a *Giardia* organism in a rabbit, which he named *Hexamita duodenalis* (Davaine 1875). Fourteen years later, the Italian protistologist Giovanni Battista Grassi (1854− 1825) published a report describing a *Giardia* species he found in a rodent (Grassi 1879). He named the organisms *Dimorphus muris*, but changed it to *Megastoma entericum* two years later (Grassi 1881). In 1882, the French botanist, zoologist, mycologist, and parasitologist Joseph Künstler (1855–1932) reported an organism in tadpoles that he named *Giardia agilis* (Künstler 1882). This was the first time *Giardia* was used as a genus name (in honour of the French zoologist Alfred Mathieu Giard (1846–1908)) and which settled the correct generic name. In 1888, the French physician and naturalist Raphaël Anatole Émile Blanchard (1857–1919) suggested naming the parasite *Lamblia intestinalis* (Blanchard 1888). Four years later, the American parasitologist Charles Wardell Stiles (1867–1941) changed the name to *Lamblia duodenalis* (Stiles 1902). In 1915, the American zoologist Charles Atwood Kofoid (1865–1947) and the Danish botanist and phycologist Elizabeth Bohn Christiansen (1876–1940) proposed *Giardia lamblia* instead of *Lamblia intestinalis* (Kofoid and Christiansen 1915a). However, in 1920, Kofoid reasoned that *Giardia enterica* has priority over *Lamblia intestinalis* and therefore the species name *Giardia lamblia* should be used for the *Giardia* parasite of man (Kofoid 1920). Eventually, in 1952, the American biologist Francis Patrick (Pasqual) Filice (1922–2015) proposed that only three species, *G. duodenalis*, *G. muris*, and *G. agilis* should be considered based on the shape (pyriform, round, and slender, respectively) and average size of their trophozoites (12–15 × 6–8 μm, 9–12 × 5–7 μm, and 20–30 × 4–5 μm, respectively) (Filice 1952; Monis et al. 2009). Concerning the human-infectious *Giardia* organism, the species name *G. lamblia* was widely used in the 1970 s, while *G. duodenalis* was used in the 1980 s, and *G. intestinalis* in the 1990 s (Adam 2001).

A detailed description of the cytological morphology of *G. duodenalis* trophozoites and cysts was published by the American medical zoologist Charles Edmund Simon (1866–1927) in 1921 (Simon 1921). Already in 1915, Kofoid and Christiansen described the fundamentals of the life cycle of *Giardia* sp. using the rodent species *G. microti* as an example (Kofoid and Christiansen 1915b). However, the pathogenicity of *G. duodenalis* to humans was not established until 1987 when Nash and colleagues demonstrated that the parasite causes disease in volunteers after inoculation of trophozoites (Nash et al. 1987).

## Outbreaks in the recent past

During the 20th century, there were repeated outbreaks of giardiasis in industrialised nations, most of which were associated with contaminated drinking water (Krumrie et al. 2022). A notable outbreak of protracted, intermittent diarrhoea caused by *G. duodenalis* occurred in winter sports enthusiasts at an Aspen ski resort in Colorado, USA, in the 1965–1966 ski season (Moore et al. 1969). Remarkably, another giardiasis outbreak happened in the Aspen ski resort region in 1981 (Instre et al. 1984). In both cases, there was strong evidence that the disease was spread by contaminated municipal tap water. Overall, there were 242 outbreaks (41000 cases) of giardiasis in the USA between 1971 and 2011 (Adam et al. 2016). A large community outbreak of giardiasis with a total of 1300 laboratory-confirmed cases occurred in the Norwegian city of Bergen in October 2004 (Nygård et al. 2006). Further investigations found that the outbreak was caused by leaking sewage water pipes in combination with poor water treatment. Another example is the outbreak in a municipality of the Bologna province in Italy from November 2018 to April 2029 with 199 confirmed cases of giardiasis (Resi et al. 2021). Tap water was most likely the source of the infection. The examples given support the numerous studies that show that outbreaks of giardiasis in modern society are mainly due to the spread of *G. duodenalis* through water supply and disposal systems. It should also be noted that in addition to outbreaks, sporadic infections play an important role in the transmission of giardiasis, too.

## *Giardia duodenalis* genetic assemblages

From the beginning, the taxonomy of *G. duodenalis* was unclear and confusing. The reason for this is the uniform morphology of *Giardia* parasites sampled from a wide host range. By the 1980 s, it became clear that *G. duodenalis* is a species complex comprising several cryptic species. By using restriction fragment length polymorphism (RFLP) and Southern blot analysis of 15 *G. duodenalis* isolates, Nash and coworkers were able in 1985 to distinguish three different groups of genotypes, with one group being significantly distinct from the other two groups (Nash et al. 1985). Four years later, allozyme electrophoresis studies revealed the presence of four discrete genetic groups within samples of 29 Australasian stocks and 48 clones of *G. duodenalis* from humans (Andrews et al. 1989). Based on further allozyme analysis, Mayrhofer and colleagues suggested in 1995 to group *G. duodenalis* isolates from humans into two major genetic assemblages, termed A and B (Mayrhofer et al. 1995). These two assemblages comprise four genetic groups: while assemblage A consists of Nash groups 1 and 2 and Mayrhofer groups 1 and 2, assemblage B includes Nash group 3 and Mayrhofer groups 3 and 4. This division of *G. duodenalis* into assemblages A and B was confirmed one year later by nucleotide sequence analysis of a 690-bp stretch of the glutamate dehydrogenase (gdh) gene (Monis et al. 1996). In 1998, two additional assemblages, C and D, were identified by genetic analysis of *G. duodenalis* isolates obtained from dogs (Monis et al. 1998). Three more *G. duodenalis* assemblages, E (hoofed livestock), F (cat), and G (rat), were established by molecular systematics in 1999 (Monis et al. 1999). A final assemblage H was introduced in 2010 for *G. duodenalis* isolates from a grey seal and a gull (Lasek-Nesselquist et al. 2010). Eventually, a taxonomic revision of the *G. duodenalis* species complex, including new species names, species descriptions, and host associations, was proposed in 2023 (Table 1) (Wielinga et al. 2023).

**Table 1:** Summary of the taxonomic description of the *G. duodenalis* species complex according to Wielinga et al. (2023)

Assemblage	Description of type specimen	Whole genome sequence	Differentiation by gdh locus substitutions^a^	Host range	New proposed name^b^
AI	Davaine 1875	AACB00000000	TGCCAGGCCTTT; TCCCTTTCC	broad	*G. duodenalis*
AII	Lambl 1859	AHGT00000000	TGCCAGGCCTTT; CTTTCCCTT	> 90% humans	*G. intestinalis*
AIII	TBA	TBA	TGCCAGGCCTTT; CCCCTCCTC	> 85% cervids	*G. cervus*
B	Grassi 1881	ACGJ00000000; AHHH00000000	CGCCAGGCCTCG	broad	*G. enterica*
C	Hegner 1922	GCA_902209425; GCA_902221515	CACCAGGCCATT	> 85% canids (in domestic setting)	*G. canis*
D	TBA	GCA_902221465; GCA_902221485	CACCAGACCATT	> 90% canids (in wild setting)	*G. lupus*
E	Fantham 1921	ACVC00000000	TGCCAGGACATT	> 85% bovids	*G. bovis*
F	Deschiens 1926	TBA	TGCCAGACTATT	> 85% felids	*G. cati*
G	Lavier 1924	TBA	TGTTAAGCCTCT	> 90% omnivorous rodents	*G. simoni*
H	TBA	TBA	CGCCTGGCCTTG	> 90% phocids	*G. pinnipedis*

^a^The *gdh* locus substitutions are from the start of the start codon and are as follows: for nonsynonymous substitutions for inter-Assemblage differentiation (first row), 604/676/731/766/769/796/829/830/832/839/841/871; for synonymous substitution for intra-Assemblage differentiation (second row), 603/753/807/831/861/867/870/894/902

^b^Names are based on host associations and the oldest available names, where available

## Drug development

From the very beginning, drug development for the treatment of giardiasis was characterised by testing existing anti-protozoal agents. However, most early developed anti-protozoan medications were ineffective against *G. duodenalis* infections. For example, the isoquinoline alkaloid emetine, which was shown to be very effective in the treatment of *E. histolytica* infections, was found to have little effect on *G. duodenalis* (Fantham 1916; Porter 1916). Other early 20th-century anti-protozoal drugs that were unsuccessfully tested in the treatment of giardiasis include organoarsenic compounds (treparsol (arsenic acid), stovarsol (acetarsol), carbarsone, and neosalvarsan), bismuth compounds (bistovol (bismuth/stovarsol) and bismuth subgallate), iodoxyquinoline sulfonic acid (Yatren), and methylene blue (Chopra et al. 1939; Morrison and Swalm 1939).

**Fig. 7:**
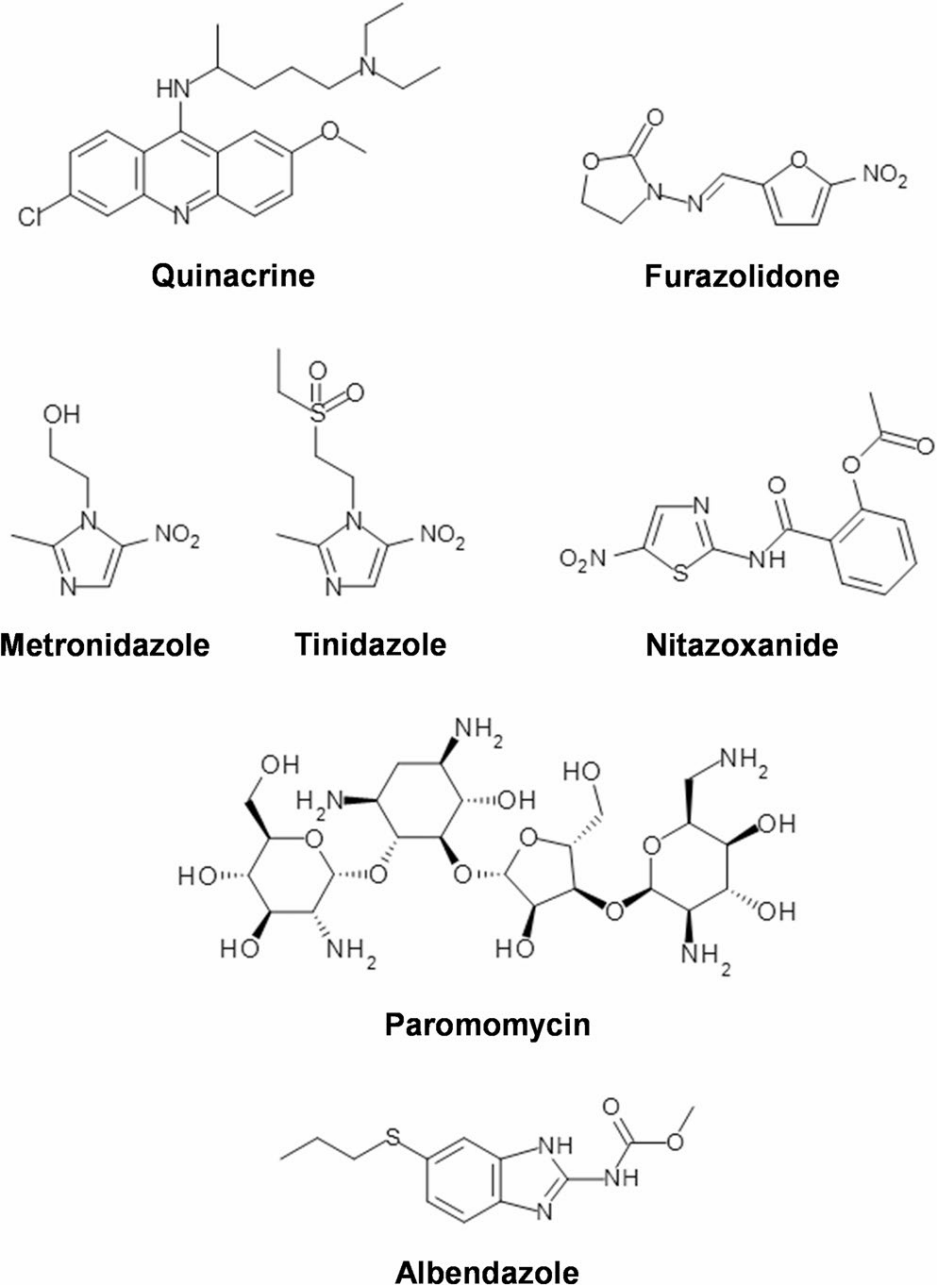
Chemical structures of drugs currently used for the treatment of human giardiasis

In 1937, the French malariologist Lucien Brumpt (1910–1999) discovered that the anti-malaria drug quinacrine (mepacrine, Fig. 7) could cure 80% of *G. duodenalis*-infected mice when given orally for 5 days (Brumpt 1937). Subsequently, several reports were published proclaiming that quinacrine is an effective drug for treating giardiasis (Martin 1937; Galli-Valerio 1937; Bacigalupo 1937; Chopra et al. 1939). Although quinacrine was the first effective drug for treating giardiasis with a reported cure rate of 92–95% (Wolfe and Handler 1998), it was withdrawn from the commercial market by the manufacturer in 1998 at the order of the FDA (Mineno and Avery 2003). Nowadays, quinacrine is reserved for the treatment of patients with diarrhoeal giardiasis who are not responding to the standard therapy (Petri 2005).

The 1960s saw the approval of three drugs for the treatment of giardiasis. The first drug to have been shown effective against *G. duodenalis* infection was the nitrofuran furazolidone (Fig. 7) (Webster 1960). The medication was developed in the 1940s and initially used for the treatment of acute bacterial diarrhoea syndrome (Ponce de Leon 1957). Furazolidone currently serves as a drug of second choice, reserved for refractory giardiasis cases (Petri 2005). The nitroimidazole metronidazole (Fig. 7) is the second medication that was found to be active against *G. duodenalis* (Schneider 1961; Rubio and Cuello 1963). The therapeutic effectiveness of metronidazole was first established in 1960 against the related protozoan parasite *Trichomonas vaginalis* (Durel et al. 1960). Since its discovery, metronidazole and its derivative tinidazole (Fig. 7), as well as the related nitrothiazole nitazoxanide (Fig. 7), are the first-line treatments for giardiasis to this day (Petri 2005). The third therapeutic agent shown to display efficacy in the treatment of giardiasis is the aminoglycoside antibiotic paromomycin (Lanzo et al. 1962; Dobón et al. 1963; Gomez Lus et al. 1964) (Fig. 7). The drug was first isolated in 1956 and subsequently approved for treatment of entamoebiasis and trichomoniasis (Gardner, and Hill, 2001). Paromomycin is currently recommended for the treatment of pregnant women with symptomatic infections of *G. duodenalis* (Petri 2005).

Originally developed as veterinary anthelminthics, the two benzimidazoles albendazole (Fig. 7) and mebendazole, have been later trialled in the treatment of giardiasis. Albendazole was found to be effective in treating children infected with *G. duodenalis*, with cure rates of 95–97% (Hall and Nahar 1993; Dutta et al. 1994). In contrast, mebendazole gave mixed results in clinical trials. While some studies reported the effectiveness of mebendazole on *G. duodenalis* infections in children (Sadjjadi et al. 2001; Escobedo et al. 2003), other studies showed an increase in prevalence of the protozoan in children during deworming programmes using the drug (Rousham 1994; Northrop-Clewes et al. 2001). Currently, only albendazole is indicated for the treatment of individuals coinfected with *G. duodenalis* and *Ascaris lumbricoides* (giant roundworm) and/or *Trichuris trichiura* (whipworm) (Petri 2005).

### Current situation

The most recent data on the incidence of giardiasis comes from the European Centre for Disease Prevention and Control (ECDC) for the European Economic Area (EEA) for 2022 (ECDC 2024). Of the 30 EEA countries, 24 reported a total of 10,894 cases of giardiasis, which corresponds to an incidence rate of 3.9 cases per 100,000 inhabitants. The highest number of giardiasis cases was registered by Spain (3298 confirmed cases), while the highest incidence rate was recorded by Luxembourg (18.9 cases/100000). Regarding age groups, children under the age of 4 years had the highest incidence rate (14.0 cases/100000). Whereas the majority of reported cases (76%) were domestically acquired, 73% and 100% of *G. duodenalis* infections in Sweden and Iceland were travel-associated, respectively.

In the period from 2011 to 2018, Scotland recorded 1631 cases of giardiasis (Ferguson et al. 2020). The mean incidence rate over the 8-year period was determined to be 3.8 cases/100,000, which is similar to the incidence rate of the EEA countries for 2022. In contrast to the EEA countries, the highest incidence rate was observed in the 20 to 49-year-old age group.

For the USA, a total of 435,186 giardiasis cases were reported during 1995–2016 (Coffey et al. 2021). Over the 22 years, the incidence rate decreased from 13.8 cases/100,000 in 1995 to 6.4 cases/100,000 in 2016. Based on 2016 data, the giardiasis incidence rate in the USA appears to be about 1.7 times higher than that in the EEA countries and Scotland. Similar to the EEA nations, children under the age of 4 years were also found to have the highest giardiasis rates in the USA throughout the study period. Whereas no outbreaks of giardiasis were registered for EEA countries (ECDC 2024), 5.1% of reported giardiasis cases in the USA were outbreak-associated from 1995 to 2016.

Compared to developed nations, the giardiasis prevalence rate in low-income countries is much higher, ranging from 10% to 50% (Gutiérrez 2017). Especially, indigenous children in developing countries are affected by the infectious disease. For example, a review of 22 studies conducted between 2011 and 2020 investigating the prevalence of giardiasis among the Malaysian population found that the overall mean rate of giardiasis was 13.7% while the prevalence in indigenous schoolchildren was with 23.1% (range: 15.1–34.6%) much higher (Roshidi et al. 2021). A similar giardiasis prevalence rate of 27.1% was reported for pupils attending three rural elementary schools in Loka Abaya town, Sidama zone, Ethiopia, between December 2018 and July 2019 (Hajare et al. 2022). In addition, the studies showed that the much higher giardiasis infection rate in developing countries is mainly due to poor hygienic lifestyle and unsatisfactory sanitation, particularly in rural regions with inadequate access to clean water and proper waste disposal.

The Global Enteric Multicenter Study (GEMS) found no positive association between giardiasis and moderate-to-severe diarrhoea in children under the age of 5 in developing countries (Kotloff et al. 2013). On the contrary, *G. duodenalis* infections were significantly less frequently identified in paediatric patient aged 1–5 years with moderate-to-severe diarrhoea than in matched controls. In a follow-up study with Israeli Arab preschool children it was found that giardiasis even may protect against diarrhoeal disease (Muhsen et al. 2014).

Giardiasis has also a significant economic impact in high-income countries. For instance, the total economic costs of the disease (medical costs, monetised Quality-Adjusted Life Year losses, and illness-related mortality) in the USA in 2010 was US$ 282 million (Devleesschauwer et al. 2017). However, the Disability-Adjusted Life Years (DALYs) attributable to giardiasis are rather low in industrialised nations. For example, the giardiasis-associated DALYs in the European region were estimated at 0.03 (UI: 0.009–0.1.009.1) per 100,000 population in 2010 (Torgerson et al. 2015). In contrast, low-income countries have much higher DALYs caused by *G. duodenalis*. For instance, the African region had a giardiasis-associated DALYs of 0.8 (UI: 0.2–3.2) per 100,000 population in 2010, 27-times higher compared to the European region (Torgerson et al. 2015).

## Conclusion

Although *G. duodenalis* branches at the base of the eukaryotic tree in phylogenetic analysis, the protozoan parasite is not a primitive eukaryote lacking canonical eukaryotic organelles. In fact, the lack or reduction of typical eukaryotic organelles and metabolic pathways in *G. duodenalis* has arisen through secondary loss and adaptation to a parasitic lifestyle. Moreover, the analysis of the genome of *G. duodenalis* confirmed that the protozoan has diverged from higher eukaryotes after the endosymbiosis of mitochondria and is not a living amitochondrial eukaryotic relic.

Since *G. duodenalis* was detected in specimens dating back to pre-Columbian times, it is most likely that the parasite was introduced into the Americas by the Palaeolithic hunter-gatherers peopling the continent some 15,000 years ago. Although giardiasis usually lasts only a few weeks, person-to-person transmission and autoinfection can lead to persistent infections. The ability to cause long-lasting infections could explain why the parasite can remain in a group of people during their migration across Beringia into the New World. Thus, *G. duodenalis* has probably been a parasite of humans for a long time (> 15,000 years), suggesting that the flagellate may be an heirloom parasite.

Although it seemed that *G. duodenalis* was established as a species by the mid-20th century, it soon became clear that the protozoan parasite is a species complex consisting of phylogenetically distinct genotypes. By using an array of molecular techniques, it was eventually possible to distinguish 8–11 different assemblages and sub-assemblages. Most recently, an analysis of thousands of published genotyping data of *G. duodenalis* isolates, together with a comprehensive review of host associations and molecular species testing, led to the proposition of new descriptions and names of *Giardia* species types infecting specific hosts.

While the prevalence of giardiasis in high-income countries is normally low, the disease can affect travellers visiting areas with poor sanitation standards. In addition, giardiasis remains a public health concern in many low-income countries, particularly in schoolchildren living in rural regions.

## Data Availability

The author declares that data supporting the findings of this study are available within the article.
